# Methyl 5,6-dimeth­oxy-1*H*-indole-2-carboxyl­ate

**DOI:** 10.1107/S1600536809034667

**Published:** 2009-09-05

**Authors:** Thomas Blake Monroe, Yasamin Moazami, Daniel S. Jones, Craig A. Ogle

**Affiliations:** a Department of Chemistry, The University of North Carolina at Charlotte, 9201 University City Blvd, Charlotte, NC 28223, USA

## Abstract

The title compound, C_12_H_13_NO_4_, was prepared as a precursor to an indole derivative with possible anti­mitotic properties. The mol­ecule is very nearly planar; the maximum deviation of any non-H atom from the mean plane of the indole ring is 0.120 (3) Å for each of two meth­oxy C atoms. The pairs of mol­ecules related by the inversion centre at (0,0,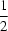) are connected by two symmetry-equivalent N—H⋯O hydrogen bonds, while the pairs of mol­ecules related by the inversion centre at (0,0,0) exhibit a π-stacking inter­action of the indole rings, with an inter­planar separation of 3.39 (3) Å.

## Related literature

For related structures see: Shoja (1988*a* ▶,*b* ▶). For pharmaceutical applications see: Fuwa & Sasaki (2009 ▶); Li & Martins (2003 ▶). For a study of π–π packing inter­actions see: Janiak (2000 ▶).
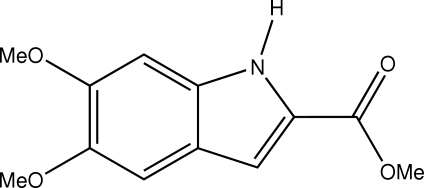


## Experimental

### 

#### Crystal data

C_12_H_13_NO_4_
*M* *_r_* = 235.23
Orthorhombic, 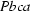

*a* = 17.0768 (19) Å
*b* = 7.7232 (11) Å
*c* = 17.678 (2) Å
*V* = 2331.5 (5) Å^3^
*Z* = 8
Cu *K*α radiation
μ = 0.85 mm^−1^
*T* = 295 K
0.36 × 0.22 × 0.21 mm

#### Data collection

Enraf–Nonius CAD-4 diffractometer
Absorption correction: none
9909 measured reflections
2098 independent reflections
1522 reflections with *I* > 2σ(*I*)
*R*_int_ = 0.030
3 standard reflections every 171 reflections intensity decay: 1%

#### Refinement

*R*[*F* ^2^ > 2σ(*F* ^2^)] = 0.035
*wR*(*F* ^2^) = 0.102
*S* = 1.02
2098 reflections
162 parameters
H atoms treated by a mixture of independent and constrained refinement
Δρ_max_ = 0.14 e Å^−3^
Δρ_min_ = −0.12 e Å^−3^

### 

Data collection: *CAD-4 EXPRESS* (Enraf–Nonius, 1994 ▶); cell refinement: *CAD-4 EXPRESS*; data reduction: *XCAD4* (Harms & Wocadlo, 1995 ▶); program(s) used to solve structure: *SHELXS97* (Sheldrick, 2008 ▶); program(s) used to refine structure: *SHELXL97* (Sheldrick, 2008 ▶); molecular graphics: *ORTEP-3 for Windows* (Farrugia, 1997 ▶) and *Mercury* (Macrae *et al.*, 2006 ▶); software used to prepare material for publication: *WinGX* (Farrugia, 1999 ▶).

## Supplementary Material

Crystal structure: contains datablocks global, I. DOI: 10.1107/S1600536809034667/fj2241sup1.cif

Structure factors: contains datablocks I. DOI: 10.1107/S1600536809034667/fj2241Isup2.hkl

Additional supplementary materials:  crystallographic information; 3D view; checkCIF report

## Figures and Tables

**Table 1:** Hydrogen-bond geometry (Å, °)

*D*—H⋯*A*	*D*—H	H⋯*A*	*D*⋯*A*	*D*—H⋯*A*
N—H1⋯O1^i^	0.926 (19)	2.011 (19)	2.867 (2)	152.9 (16)

Symmetry code: (i) 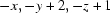.

**Fig. 1.**
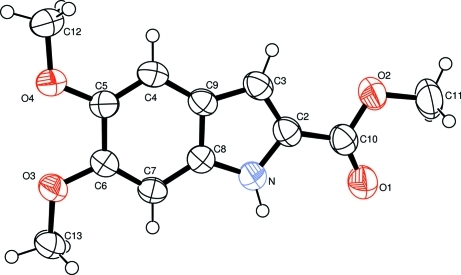
View of the title compound (50% probability displacement ellipsoids).

**Fig. 2.**
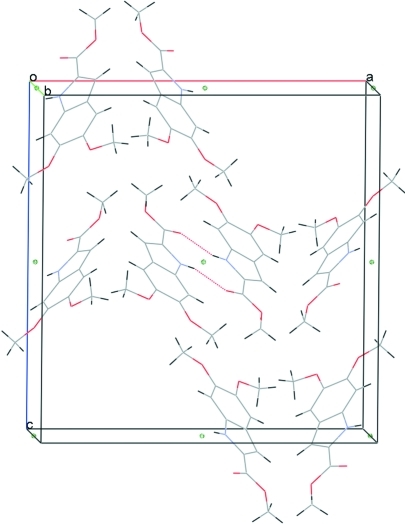
Packing diagram showing the hydrogen bonding between molecules related by inversions at (0,0,1/2).

**Fig. 3.**
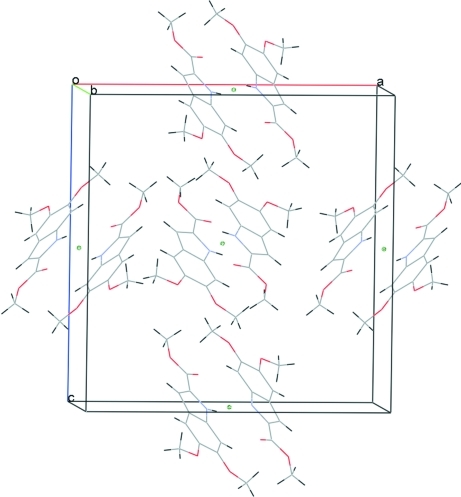
Packing diagram showing the π–π interactions between molecules related by inversions at (0,0,0).

**Fig. 4.**
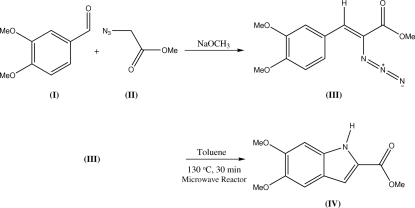
Synthesis scheme

## supplementary crystallographic information

### Comment

The indole core is a common structure observed in a wide variety of biologically active compounds and pharmaceutical products (Li & Martins, 2003). Indole structures are considered as privileged structure motifs, due to their ability to bind many receptors within the body (Fuwa & Sasaki, 2009). As a result, a great deal of research has been dedicated to incorporating the indole functionality in the design and synthesis of anti-mitotic compounds for the treatment of cancer. The title compound was prepared as a precursor to an indole derivative with possible anti-mitotic properties.

The title molecule is nearly planar; the deviations of the methoxy carbons from the indole mean plane are 0.058 (3) Å, 0.119 (3) Å, and -0.120 (3) Å for C13, C12, and C11, respectively. These values can be compared with those for two similar structures. In 5,6-Dimethoxyindole (Shoja, 1988*a*) one of the methoxy carbon atoms was out of the plane by 0.257 (4) Å, while in 5,6-Dimethoxy-1-indanone (Shoja, 1988*b*) one of the methoxy carbon atoms was out of the plane of the aromatic ring by 0.270 Å.

The members of each pair of molecules related by inversions at (0,0,1/2) are joined by two symmetry-equivalent N—H···O hydrogen bonds, as shown in Figure 2 and described in Table 1. The indole ring system of each pair of molecules related by inversions at (0,0,0) exhibit π-π interactions, as shown in Figure 3. An exhaustive study has been made of structures in the Cambridge Structural Database which show π-π interactions between nitrogen-containing aromatic ring systems (Janiak, 2000). This study showed that parallel ring systems which interact are offset by an amount related to the distance between ring centroids. The planes of the indole rings of the present structure are 3.39 (3) Å apart, and the centroid-centroid line makes an angle of 23.8° with the normal to the plane of the indole rings. These values are in agreement with those found for similar systems in the Janiak study. The π-π interactions may account for the near-planarity of the molecule.

### Experimental

Preparation of title compound (IV): In a two-necked round-bottomed flask containing 2 ml of methanol, 244 mg (1.47 mmol) of 3,4-dimethoxybenzaldehyde (I) (commercially available) and 575 mg (5.0 mmol) of methyl 2-azidoacetate (II) were dissolved under N_2_. This solution was cooled to 0 °C in an ice bath. Freshly prepared NaOMe in methanol was added to the mixture of the aldehyde and the azide compounds drop-wise over 15 minutes. The mixture gradually formed a slurry upon reacting with the NaOMe. The reaction was further stirred for 2.5 h and then poured into 50 ml of water. This resulted in the formation of a solid yellow precipitate (III) which was separated from the liquid by suction filtration. The solid (III) was then dissolved in 3 ml of toluene and transferred to a clean, dry microwave reactor vessel equipped with a stir bar. The vessel was sealed with a septum and heated in the microwave reactor at 130 °C for 30 minutes. At the end of heating the vessel was purged with a needle to release the gas pressure. The final product (IV) crystallized from the toluene and was separated by suction filtration in 70% yield.

### Refinement

H1, the hydrogen atom bonded to N, was located in a difference map and refined. All other H atoms were constrained using a riding model. The aromatic C—H bond lengths were fixed at 0.93 Å and the methyl C—H bond lengths at 0.96 Å, with *U*_iso_(H) = 1.5 *U*_eq._ (C). An idealized tetrahedral geometry was used for the methyl groups, and the torsion angles around the O—C bonds were refined.

### Crystal data

**Table:**

C_12_H_13_NO_4_	*F*(000) = 992
*M**_r_* = 235.23	*D*_x_ = 1.34 Mg m^−^^3^
Orthorhombic, *P**b**c**a*	Cu *K*α radiation, λ = 1.54184 Å
Hall symbol: -P 2ac 2ab	Cell parameters from 25 reflections
*a* = 17.0768 (19) Å	θ = 10.0–43.0°
*b* = 7.7232 (11) Å	µ = 0.85 mm^−^^1^
*c* = 17.678 (2) Å	*T* = 295 K
*V* = 2331.5 (5) Å^3^	Prism, colourless
*Z* = 8	0.36 × 0.22 × 0.21 mm

### Data collection

**Table:**

Enraf–Nonius CAD-4 diffractometer	θ_max_ = 67.4°, θ_min_ = 5°
Non–profiled ω/2θ scans	*h* = −20→20
9909 measured reflections	*k* = 0→9
2098 independent reflections	*l* = −21→21
1522 reflections with *I* > 2σ(*I*)	3 standard reflections every 171 reflections
*R*_int_ = 0.030	intensity decay: 1%

### Refinement

**Table:**

Refinement on *F*^2^	H atoms treated by a mixture of independent and constrained refinement
Least-squares matrix: full	*w* = 1/[σ^2^(*F*_o_^2^) + (0.0613*P*)^2^ + 0.2248*P*] where *P* = (*F*_o_^2^ + 2*F*_c_^2^)/3
*R*[*F*^2^ > 2σ(*F*^2^)] = 0.035	(Δ/σ)_max_ < 0.001
*wR*(*F*^2^) = 0.102	Δρ_max_ = 0.14 e Å^−^^3^
*S* = 1.02	Δρ_min_ = −0.12 e Å^−^^3^
2098 reflections	Extinction correction: *SHELXL97* (Sheldrick, 2008), Fc^*^=kFc[1+0.001xFc^2^λ^3^/sin(2θ)]^-1/4^
162 parameters	Extinction coefficient: 0.0047 (4)
0 restraints	

### Special details

**Table:**

Geometry. All s.u.'s (except the s.u. in the dihedral angle between two l.s. planes) are estimated using the full covariance matrix. The cell s.u.'s are taken into account individually in the estimation of s.u.'s in distances, angles and torsion angles; correlations between s.u.'s in cell parameters are only used when they are defined by crystal symmetry. An approximate (isotropic) treatment of cell s.u.'s is used for estimating s.u.'s involving l.s. planes.

### Fractional atomic coordinates and isotropic or equivalent isotropic displacement parameters (Å^2^)

**Table:**

	*x*	*y*	*z*	*U*_iso_*/*U*_eq_	
N	0.05760 (8)	0.76815 (19)	0.51504 (7)	0.0509 (3)	
O1	0.07015 (8)	1.05091 (17)	0.41553 (7)	0.0727 (4)	
O2	0.17099 (7)	0.92154 (18)	0.35786 (7)	0.0722 (4)	
O3	0.00702 (6)	0.30330 (14)	0.69682 (6)	0.0564 (3)	
O4	0.12332 (7)	0.14080 (15)	0.64271 (7)	0.0656 (4)	
C2	0.11320 (9)	0.7786 (2)	0.45863 (8)	0.0516 (4)	
C3	0.15832 (9)	0.6316 (2)	0.46040 (8)	0.0530 (4)	
H3	0.2001	0.6062	0.4285	0.064*	
C4	0.15041 (8)	0.3618 (2)	0.54858 (8)	0.0495 (4)	
H4	0.1918	0.2997	0.5277	0.059*	
C5	0.10882 (9)	0.2958 (2)	0.60783 (8)	0.0476 (4)	
C6	0.04402 (8)	0.3882 (2)	0.63953 (8)	0.0453 (4)	
C7	0.02331 (8)	0.5479 (2)	0.61286 (8)	0.0459 (4)	
H7	−0.018	0.6096	0.634	0.055*	
C8	0.06679 (8)	0.6150 (2)	0.55251 (8)	0.0448 (4)	
C9	0.12971 (8)	0.5256 (2)	0.51953 (8)	0.0466 (4)	
C10	0.11447 (10)	0.9300 (2)	0.41017 (9)	0.0556 (4)	
C11	0.17434 (13)	1.0657 (4)	0.30566 (12)	0.0936 (8)	
H11A	0.1271	1.0693	0.2763	0.14*	
H11B	0.2184	1.0517	0.2725	0.14*	
H11C	0.1797	1.1718	0.3335	0.14*	
C12	0.18869 (10)	0.0457 (2)	0.61719 (11)	0.0661 (5)	
H12A	0.1826	0.0201	0.5644	0.099*	
H12B	0.1925	−0.0604	0.6452	0.099*	
H12C	0.2354	0.1128	0.6246	0.099*	
C13	−0.05334 (10)	0.3942 (2)	0.73565 (10)	0.0622 (5)	
H13A	−0.0324	0.4989	0.7568	0.093*	
H13B	−0.0737	0.3228	0.7755	0.093*	
H13C	−0.0946	0.4221	0.7009	0.093*	
H1	0.0202 (10)	0.852 (2)	0.5251 (10)	0.064 (5)*	

### Atomic displacement parameters (Å^2^)

**Table:**

	*U*^11^	*U*^22^	*U*^33^	*U*^12^	*U*^13^	*U*^23^
N	0.0517 (7)	0.0518 (8)	0.0492 (7)	−0.0002 (6)	0.0024 (6)	0.0013 (6)
O1	0.0850 (9)	0.0656 (9)	0.0676 (8)	0.0067 (7)	0.0120 (7)	0.0111 (6)
O2	0.0623 (7)	0.0919 (10)	0.0624 (7)	0.0005 (7)	0.0096 (6)	0.0236 (7)
O3	0.0601 (6)	0.0515 (7)	0.0576 (6)	0.0056 (5)	0.0185 (5)	0.0030 (5)
O4	0.0607 (7)	0.0554 (7)	0.0806 (8)	0.0140 (6)	0.0226 (6)	0.0125 (6)
C2	0.0472 (8)	0.0623 (10)	0.0454 (8)	−0.0074 (8)	−0.0004 (7)	0.0008 (7)
C3	0.0422 (8)	0.0693 (11)	0.0475 (8)	−0.0042 (8)	0.0021 (6)	0.0007 (8)
C4	0.0390 (7)	0.0577 (10)	0.0517 (8)	0.0014 (7)	0.0033 (6)	−0.0050 (8)
C5	0.0442 (8)	0.0465 (9)	0.0520 (8)	0.0006 (6)	0.0013 (6)	−0.0026 (7)
C6	0.0437 (7)	0.0482 (9)	0.0438 (7)	−0.0035 (7)	0.0026 (6)	−0.0036 (7)
C7	0.0435 (8)	0.0486 (9)	0.0457 (8)	0.0003 (7)	0.0029 (6)	−0.0069 (7)
C8	0.0431 (7)	0.0474 (8)	0.0438 (7)	−0.0037 (6)	−0.0037 (6)	−0.0030 (7)
C9	0.0375 (7)	0.0583 (10)	0.0441 (7)	−0.0041 (7)	−0.0016 (6)	−0.0028 (7)
C10	0.0522 (9)	0.0675 (11)	0.0470 (8)	−0.0070 (9)	−0.0033 (7)	0.0026 (8)
C11	0.0829 (14)	0.124 (2)	0.0739 (12)	−0.0071 (13)	0.0069 (11)	0.0451 (13)
C12	0.0582 (10)	0.0595 (11)	0.0806 (12)	0.0141 (9)	0.0098 (9)	0.0036 (9)
C13	0.0647 (10)	0.0622 (10)	0.0598 (10)	0.0076 (9)	0.0219 (8)	0.0008 (9)

### Geometric parameters (Å, °)

**Table:**

N—C8	1.365 (2)	C4—H4	0.93
N—C2	1.3792 (19)	C5—C6	1.431 (2)
N—H1	0.926 (19)	C6—C7	1.367 (2)
O1—C10	1.206 (2)	C7—C8	1.399 (2)
O2—C10	1.338 (2)	C7—H7	0.93
O2—C11	1.447 (2)	C8—C9	1.404 (2)
O3—C6	1.3619 (18)	C11—H11A	0.96
O3—C13	1.4235 (19)	C11—H11B	0.96
O4—C5	1.3695 (19)	C11—H11C	0.96
O4—C12	1.410 (2)	C12—H12A	0.96
C2—C3	1.373 (2)	C12—H12B	0.96
C2—C10	1.449 (2)	C12—H12C	0.96
C3—C9	1.415 (2)	C13—H13A	0.96
C3—H3	0.93	C13—H13B	0.96
C4—C5	1.364 (2)	C13—H13C	0.96
C4—C9	1.410 (2)		
			
C8—N—C2	108.82 (14)	C7—C8—C9	122.74 (14)
C8—N—H1	126.2 (11)	C8—C9—C4	118.80 (14)
C2—N—H1	125.0 (11)	C8—C9—C3	106.66 (14)
C10—O2—C11	115.57 (16)	C4—C9—C3	134.55 (14)
C6—O3—C13	117.19 (12)	O1—C10—O2	123.01 (16)
C5—O4—C12	117.03 (13)	O1—C10—C2	124.67 (15)
C3—C2—N	108.74 (14)	O2—C10—C2	112.32 (16)
C3—C2—C10	132.24 (14)	O2—C11—H11A	109.5
N—C2—C10	119.01 (15)	O2—C11—H11B	109.5
C2—C3—C9	107.56 (14)	H11A—C11—H11B	109.5
C2—C3—H3	126.2	O2—C11—H11C	109.5
C9—C3—H3	126.2	H11A—C11—H11C	109.5
C5—C4—C9	118.94 (14)	H11B—C11—H11C	109.5
C5—C4—H4	120.5	O4—C12—H12A	109.5
C9—C4—H4	120.5	O4—C12—H12B	109.5
C4—C5—O4	125.31 (14)	H12A—C12—H12B	109.5
C4—C5—C6	121.15 (15)	O4—C12—H12C	109.5
O4—C5—C6	113.54 (13)	H12A—C12—H12C	109.5
O3—C6—C7	124.82 (13)	H12B—C12—H12C	109.5
O3—C6—C5	114.20 (13)	O3—C13—H13A	109.5
C7—C6—C5	120.98 (14)	O3—C13—H13B	109.5
C6—C7—C8	117.36 (13)	H13A—C13—H13B	109.5
C6—C7—H7	121.3	O3—C13—H13C	109.5
C8—C7—H7	121.3	H13A—C13—H13C	109.5
N—C8—C7	129.03 (14)	H13B—C13—H13C	109.5
N—C8—C9	108.22 (13)		

### Hydrogen-bond geometry (Å, °)

**Table:**

*D*—H···*A*	*D*—H	H···*A*	*D*···*A*	*D*—H···*A*
N—H1···O1^i^	0.926 (19)	2.011 (19)	2.867 (2)	152.9 (16)

Symmetry codes: (i) −*x*, −*y*+2, −*z*+1.
